# Heart of America Annual Survey: A Call for Unity and the Power of Racial Healing

**DOI:** 10.1089/heq.2023.29041.nche

**Published:** 2024-01-12

**Authors:** Gail Christopher, Susan Eaton, Mark Herring, Heather McGhee, Brian Smedley, Michael R. Wenger

**Affiliations:** ^1^ Executive Director, National Collaborative for Health Equity, Washington, District of Columbia, USA.; ^2^ Professor of Practice and Director, Sillerman Center for the Advancement of Philanthropy, Brandeis University's Heller School for Social Policy, Waltham, Massachusetts, USA.; ^3^ Former Attorney General of Virginia.; ^4^ Brooklyn, New York, USA.; ^5^ Equity Scholar, Senior Fellow, Urban Institute, Washington, District of Columbia, USA.; ^6^ Senior Fellow, American Association of Colleges & Universities, Washington, District of Columbia, USA.

***Preface:* Bending the Arc Toward Health Equity and Justice *by Gail C. Christopher***

Those in the field of social justice and health equity advocacy say, as popularized by Dr. Martin Luther King Jr., “the arc of the moral universe is long, but it bends toward justice.” After decades of work in the field of health equity, the arc is clearly bending, but slowly. Racial inequities remain a feature of the U.S. health care landscape 20 years after the former Institute of Medicine (now the National Academy of Medicine) published the landmark report, *Unequal Treatment: Confronting Racial and Ethnic Disparities in Health Care*.^1^ This breakthrough publication unequivocally documented widespread racial and ethnic inequities in the quality of health care, even after accounting for access-related factors such as health insurance coverage, and yet, inequities persist. Therefore, it was not surprising that recent findings by the Kaiser Family Foundation (KFF) demonstrated that the experiences of people of color within the medical system were characterized by discrimination and perceived negative encounters. KFF conducted a nationally representative survey based on responses from over 6000 adults, providing new data on individuals' experiences with racism and discrimination and the impacts of these experiences, both broadly and within racial and ethnic groups. The good news is that the survey tapped into the voices of the populations directly affected by racial hierarchy that persists within the nation's health care delivery system. Findings that reflect the need to address continued discrimination and racism within the health care system include the following:

- Negative experiences with health care providers as well as language access challenges have consequences for health and health care use. Among adults who used health care in the past 3 years, one in four (25%) say they had a negative experience (including being treated unfairly or with disrespect, a negative provider interaction, or difficulty with language access), and it led to worse health, being less likely to seek care, and/or switching providers. American Indians and Alaska Natives (AIAN) and Black adults are more likely than White adults to say they had a negative experience, and it contributed to at least one of these consequences.
- Having providers with a shared background matters, as Black, Hispanic, and Asian adults who have more health care visits with providers who share their racial and ethnic background report more frequent positive and respectful interactions. Reflecting on the limited racial and ethnic diversity of the health care workforce, most Hispanic, Black, Asian, and AIAN adults say that fewer than half of their health care visits in the past 3 years were with a providers who shared their racial or ethnic background. However, the survey shows how provider racial and ethnic concordance can make a difference in patient interactions. For example, Black adults who had at least half of recent visits with a provider who shares their background are more likely than those who have fewer of these visits to say that their doctor explained things in a way they could understand (90% vs. 85%), involved them in decision-making about their care (84% vs. 73%), understood and respected their cultural values or beliefs (84% vs. 76%), or asked them about social and economic factors (39% vs. 24%) during recent visits.^2^

The persistence of the reported discrimination and negative encounters by people of color could be partially contributed to the lack of diversity in our health care workforce. The National Institute of Health published a study in 2019 documenting the racial/ethnic diversity of the current health care workforce and the graduate pipeline for 10 health care professions comprising a total of 148,358,252 individuals aged 20 to 65 years old who were working or searching for work and a total of 71,608,009 individuals aged 20 to 35 years old in the educational pipeline. Among the 10 professions assessed, the mean diversity index for Black people was 0.54 in the current workforce and in the educational pipeline. In 5 of 10 health care professions, representation of Black graduates was lower than representation in the current workforce (e.g., occupational therapy: 0.31 vs. 0.50). The mean diversity index for Hispanic people was 0.34 in the current workforce; it improved to 0.48 in the educational pipeline but remained lower than 0.50 in 6 of 10 professions, including physical therapy (0.33). The mean diversity index for Native American people was 0.54 in the current workforce and increased to 0.57 in the educational pipeline.^3^ Clearly, there is a need for comprehensive strategies to compensate for the lack of diversity, while at the same time there is an urgent need to increase diversity in our workforce. Innovative interventions in our health care workforce are required.

As America seeks to bring equity to communities and advance social justice, it is vital that health care, public health policy, advocacy, and budgeting decisions focus on racial equity and healing. For too long, racism has been the root cause of health disparities and inequities that have ravaged populations of color. The work of eliminating health care disparities requires a realistic understanding of the historic and contemporary factors that contribute to health care inequities. As recently as 1965, Black patients in some states could only receive care in separate and poorly funded medical facilities, where Black physicians were relegated to practice. When President Lyndon B. Johnson signed Medicare into law, more than 7000 U.S. hospitals instantly became subject to the civil rights regulations set in Title VI. These hospitals could no longer discriminate and receive federal payments for medical services. But the decades before that were periods of protracted defiance and resistance to racial and ethnic integration.^4^ This dramatic change in 1965 was fully a hundred years after the end of the civil war. That war ended more than two centuries of denial of the humanity of millions of enslaved African Americans. The permission to enslave and to deny full human and civil rights was rooted in a cultural belief in a false hierarchy of human value. This belief was certainly embedded in medical and health care practices for centuries creating a health care system that was ill equipped to address the increased vulnerability to illness precipitated by myriad social factors known today as the social determinants of health. Systemic racism and unconscious bias continue to contribute to poor health outcomes for populations of color, with far too much suffering by individuals and their families, as infants die prematurely, chronic and infectious diseases ravage communities, and many lack health insurance.

The National Collaborative for Health Equity (NCHE) has worked during the last 20 years to identify and amplify solutions to this seemingly intractable problem of health inequities for people of color. It is vital to highlight some of the innovative strategies and research efforts that are happening around the country, which can be a source for inspiration and hope.

Heart disease remains the leading killer and crippler in America.^5^ Cardiovascular disease disproportionately burdens communities of colors as well. Working with other physicians at Brigham and Women's Hospital in Boston, Dr. Michelle E. Morse spearheaded a study determining that Black and Hispanic heart failure patients treated in the hospital's emergency department were disproportionately sent to general admissions rather than the specialty cardiology unit where there are better patient outcomes.^6^ In response, her leadership led to the creation of the Healing ARC intervention and framework, a blueprint for race-conscious interventions that can eliminate racism in patient care. To achieve this, they have developed a computerized clinical decision support system (CDSS) to address a racial health inequity. This race-conscious CDSS, which will be evaluated prospectively for impact, is designed to address two antiracism goals simultaneously: clinician education through acknowledgment of the racial inequity and redress for Black and Latinx patients.^7^ This creative intervention helps to mitigate the risk of discriminatory treatment in the clinical setting by leveraging technology to reduce the risk of human error.

There is an increasing awareness of the need for effective interventions, which is demonstrated by the growing number of local jurisdictions that have declared racism to be a public health crisis or emergency, which is over 250 as of this writing.^8^ These local efforts were accompanied by a courageous statement in 2021 by the then-new Director of the Centers for Disease Control and Prevention, Dr. Rochelle Walensky. “Racism is a serious public health threat that directly affects the well-being of millions of Americans. As a result, it affects the health of our entire nation.”^9^

This nation, despite its racialized history and formation, has yet to follow the lead of over 40 other countries in the world and implement a national Truth and Reconciliation Commission (TRC) effort. However, there has been leadership by the philanthropic sector to create and support local truth efforts. W. K. Kellogg Foundation (WKKF) launched the Truth, Racial Healing, and Transformation™ (TRHT) effort in 2016 in funding partnership with several family, local, and regional philanthropic entities. TRHT, while informed by the TRC model, is a unique process designed to reflect, embrace, and address the unprecedented diversity and unparalleled racialized history of the United States. With funding from the Robert Wood Johnson Foundation (RWJF), WKKF, and de Beaumont Foundation, NCHE works with leaders across the country that are committed to improving health outcomes for communities of color by adapting the comprehensive TRHT framework to their ongoing efforts to achieve health equity. The TRHT framework includes five pillars or areas of focus; these include narrative change, racial healing and relationship building, separation, law, and economy. In 2021, in partnership with the American Public Health Association (APHA) and de Beaumont Foundation, NCHE curated briefs that documented policies and practices being implemented across the country to address racism and related social determinants of health and well-being. This set of briefs, Healing through Policy, can be accessed through NCHE, APHA, and de Beaumont's websites. As the landmark study, *Unequal Treatment*, the Healing Arc cardiovascular innovation (cited above), and the KFF research indicate that accurate data are required before effective interventions can be designed and implemented. At NCHE, we believe that data have to go beyond stating disparities and move toward shifting a narrative to create expectations of accountability for measurable progress. Brian Smedley, PhD, chief editor of *Unequal Treatment* and senior fellow at the Urban Institute, worked with leading health scholars and researchers to design the Health Opportunity and Equity (HOPE) Initiative, with funding from RWJF. The HOPE Data Initiative tracks 27 indicators of child and adult health outcomes and the key resources that produce opportunities for health and well-being. What makes the HOPE Data Initiative unique is the “distance to goal” concept or the concept of focusing on the opportunity to achieve health equity. State and/or population groups can use the HOPE Indicators as a tool to identify equity gaps, set equity goals, measure distance-to-goal, and drive equity action. With funding from the John D. and Catherine T. MacArthur Foundation and RWJF, NCHE is working with local jurisdictions in adapting the HOPE national- and state-level indicators to support their local priorities and interventions. This community of practice, Leveraging HOPE to Transform Public Health Data Systems, currently includes representatives of 22 local jurisdictions.

Despite meaningful efforts over the last four decades, the diversity of the health care workforce in the United States is abysmal. As the general population becomes more diverse, this divide is even more consequential, as the KFF survey results indicate. KFF results also emphasize the importance of doctor–patient concordance. One recent study dramatically illustrates the importance of this as it relates to what is perhaps our nation's most persistent disparity—infant mortality. In the United States, Black newborns die at three times the rate of White newborns. Research examining 1.8 million hospital births in the state of Florida between 1992 and 2015 suggest that newborn–physician racial concordance is associated with a significant improvement in mortality for Black infants. A large body of work highlights disparities in survival rates across Black and White newborns during childbirth. The researchers posit that these differences may be ameliorated by racial concordance between the physician and newborn patient. Findings suggest that when Black newborns are cared for by Black physicians, the mortality penalty they suffer, as compared with White infants, is halved. Strikingly, these effects appear to manifest more strongly in more complicated cases and when hospitals deliver more Black newborns.^10^ This study is a powerful example of how data can be used to delve into and deepen our understanding of the scale and scope of the problem and foster more tailored interventions such as the type described by the physicians at Brigham and Women's Hospital. The stark racial disparities in incidents and mortalities in COVID-19 lead RWJF to create a first of its kind National Commission to Transform Public Health Data Systems in the United States. NCHE partnered in this endeavor and facilitated the adaptation of the TRHT strategy for the commission's deliberations. As a result, the recommendations are organized around three key themes: (1) center health equity and well-being in narrative change, (2) prioritize equitable governance and community engagement, (3) ensure public health measurement captures and addresses structural racism and other inequities. With continued support from RWJF, the commission's recommendations are influencing federal policy, public health organizations, and jurisdictions across the nation. Additional encouragement comes from the fact that leading health and medical organizations have moved beyond denial of the problem and have begun the hard work of acknowledging how they have contributed to the problem and are beginning to take meaningful, corrective action. Examples include

- In June 2021, the American Medical Association's House of Delegates, representing their peers from all corners of medicine, voted to adopt guidelines addressing systemic racism in medicine, including discrimination, bias, and abuse, including expressions of prejudice known as microaggressions. AMA recommended that health care organizations and systems use new guidelines to establish institutional policies that promote positive cultural change and ensure a safe, discrimination-free work environment.^11^
- In October 2021, the American Psychological Association's Council of Representatives adopted a resolution because of their role in promoting, perpetuating, and failing to challenge racism, racial discrimination, and human hierarchy in the U.S. Recognizing that many existing historical records and narratives have been centered in Whiteness, APA also concluded that it was imperative to capture oral history and the lived experiences of communities of color, so they commissioned a series of listening sessions and surveys, which also informed their resolution.^12^
- In June 2020, the American Psychiatric Association Presidential Task Force to Address Structural Racism Throughout Psychiatry (TFSR) was formed by APA President, Dr. Jeffrey Geller. The APA Board of Trustees' Structural Racism Accountability Committee (SRAC) was formed in 2021 to ensure that the recommendations of the 2020–2021 TFSR were carried out. This resulted in 18 recommendations that were approved by the SRAC.^13^

As the diversity of our child and youth populations increases, it is also very promising that the American Academy of Pediatrics (AAP) is taking steps to address the issue of racism and racial discrimination. In 2019, AAP issued a policy statement that provided an evidence-based document focused on the role of racism in child and adolescent development and health outcomes. The intention of this policy statement was to better equip pediatricians to proactively engage in strategies to optimize clinical care, workforce development, professional education, systems engagement, and research in a manner designed to reduce the health effects of structural, personally mediated, and internalized racism and improve the health and well-being of all children, adolescents, emerging adults, and their families.^14^ Achieving the goals of overcoming the legacy of racism and providing opportunities for health and well-being for historically marginalized communities requires accelerated efforts. In a comprehensive way, the KFF research is extremely valuable because it gives the perspective of diverse populations and their lived experiences interacting with the health care delivery system. As Dr. Martin Luther King Jr. admonished us, “Of all forms of discrimination and inequalities, injustice in health is the most shocking and inhuman.”

Today, NCHE is proud to present five distinguished leaders in various disciplines important to achieving racial and health equity. This special roundtable event with Health Equity, a peer-reviewed journal that is NCHE's official publication partner, is leveraging the insights gleaned from NCHE's first Heart of America Annual Survey. This survey explores our nation's readiness for overcoming our divisions and healing from our racialized past. We are also pleased to announce the following five new briefs, background papers related to racial and health equity:

- *21st Century Narrative Change with Focus on Social Media,* written by Amy Sprecher and Aaliytha Stevens, Cofounders: Building CommUnity LLC (Supplementary Data S1)
- *Facilitating Social Transformation Through Self and Collective Healing: A Collection of Insights, Resources, and Practices,* written by Colette Rausch, Director, Neuroscience and Peacebuilding, Think Peace; and Laura Webber, Convener, Think Peace Learning and Support Hub (Supplementary Data S2)
- *Toward Transformative Reparations,* written by Rob Corcoran, Program Design & Training Consultant, Initiatives of Change International; and Mike Wenger, Senior Fellow, American Association of Colleges and Universities (AAC&U) (Supplementary Data S3)
- *Segregation Yesterday and Today: Exploring Possibilities for Systemic Change,* written by Susan Eaton, Professor of Practice and Director, The Sillerman Center, The Heller School for Social Policy and Management at Brandeis University (Supplementary Data S4)
- *The Economic Well-being of Black Americans and the Implications for Health Equity* written by Darrell J. Gaskins, PhD, MS, William C. and Nancy F. Richardson Professor in Health Policy and Director, Hopkins Center for Health Disparities Solutions, Department of Health Policy and Management, Johns Hopkins School of Management and Public Health (Supplementary Data S5)

The perspectives shared in the following roundtable discussion in the *Health Equity* journal, as well as the knowledge shared in the background papers listed above, should be of interest and value to executives, leaders, and educators in all sectors across the country. The insights shared by roundtable participants demonstrate that progress can be made in facing and overcoming our nation's historic and contemporary divisions. Their contributions and the results of NCHE's annual survey remind us that our democracy can be strengthened by increasing our individual and collective ability to see ourselves through a lens of shared humanity. We encourage you to read and share this publication.

## Supplementary Material

Supplementary Data S5

For more information see: www.nationalcollaborative.org/racial-healing

## Notes

***Dr. Gail Christopher:* The National Collaborative for Health Equity (NCHE) is a nonprofit organization based in Washington, DC, that is committed to ending racial health disparities. We have been doing this work for 20 years or more, and we recognize that we will only end health inequities and health disparities in this country when we end racism. So, we are expanding our mission to do just that, to work on ending racism in America. We recognize doing that requires a comprehensive strategy, and we have embraced the Truth, Racial Healing, and Transformation (TRHT) strategy that I created while serving as vice president and senior advisor at the W.K. Kellogg Foundation.**

**The TRHT strategy has five core pillars—narrative change, racial healing and relationship building, and then overcoming the legacy that is embedded in our systems, and that legacy was one of separation. Our legal system embeds racial hierarchies, so another pillar is the law. And certainly, racial hierarchy fueled our economy and continues to do so today. To summarize, the five pillars are narrative change, racial healing and relationship building, separation, law, and economy.**

**The first pillar, narrative change, is so important. Every major movement in our country toward social justice or equity paralleled a major shift in our media infrastructure and our nation's communications industry. Even as far back as the abolition movement that was fueled by the development and wide distribution of newspapers across America. The Civil Rights movement in the ’60s was enhanced by the development of television and radio. That movement found its way into the homes of everyday Americans. And now we are in an unprecedented era where technology platforms have driven social media and artificial intelligence, and we must find ways to leverage this revolution in communications to drive a narrative change that will, in fact, help to foster the transformation that America requires if we're going to end racism, achieve equity, and truly actualize our inherent tenets of this democracy.**

**So we at the National Collaborative have inaugurated an annual poll that we are calling the Heart of America Annual Survey.^*^ We did some research. We looked at the major polls that are being used to take the pulse of our country, and we realized that far too many of those surveys and polls are merely capturing and amplifying a dominant narrative about division and polarization.**

**Yet our elections are continuing to show us that the general electorate is not so extreme or polarized. NCHE decided to develop a survey, which we intend to do annually, to take the pulse of America's readiness to do the hard work of healing, of overcoming our legacy of racial division, and of actualizing our core democratic ideals and tenets. This first survey occurred in 2023, and there were some amazing findings, one of which is that almost a third (31%) of Americans want our nation to do the relational work that will enable us to see ourselves through a lens of shared humanity.**

**Eight out of 10 respondents recognize that diversity in the workplace is an important requirement for us overcoming our differences, and they believe that teaching our nation's history of racism in our school system is a necessary step in our healing and our overcoming our historic divides. But perhaps most encouraging, more than 80% of the respondents want a leader that unites rather than divides us. There is really a readiness in this country for us to put polarization and division behind us so that we can solve our collective and our common challenges and problems.**

**We are so pleased to have a wonderful roundtable today that is going to discuss the importance of this work in our country. And we have Dr. Brian Smedley. Brian Smedley has been very creative in his efforts over the years to help us overcome health inequities. We're pleased to have former Attorney General Mark Herring, who has been courageous in his efforts in Virginia. We also have Dr. Susan Eaton, who is a professor, activist and scholar. We have Mike Winger, an author, leader and activist for racial equity, and Heather McGhee, a renowned thought leader and author of an award winning, New York Times' Bestselling book. So together, they are going to discuss these issues, and we are so excited to bring you this roundtable with critical insights from these leaders.**

**We have learned that one of the most important tools for driving a new narrative for deepening our understanding authentically is data. How well do we use data? The COVID-19 pandemic really revealed how poorly our public health data systems were able to capture the inequities associated with COVID, but we have been in the business of transforming our approaches to data for some time. I am going to invite Brian Smedley to tell us about one of his creative efforts.**

**Dr. Smedley, can you tell us how the Health Opportunity and Equity Initiative (HOPE) data initiative, with its distance-to-goal strategy, demonstrates how data can be used to change the narrative for addressing health inequities and, at the same time, support action by policymakers and leaders?**

**Dr. Brian Smedley:** Thank you for that question, Dr. Christopher. You are so right that the ways that we measure the question of equity contributes either in positive, constructive ways to a narrative that we are trying to build, or it can contribute to a more destructive narrative. And I would argue that the way that we have measured inequity in the decades before the current crop of research has been not as helpful as some of the more recent efforts to measure progress toward advancing equity.

So, the HOPE was conceived with the idea that measuring progress toward health equity needs to, one, inspire people to action. Too often, prior disparities-oriented research was very deficit oriented. It implied that there was something wrong with populations of color or people who fared poorly on most of those measures. Secondly, many prior examples of disparities research used the White population as the normative population or the comparison group against which other populations would be compared, and it falsely suggests that, one, the white population is the healthiest population in every instance or is doing better than other populations. And it also falsely suggests that being White is the aspiration, that being White is somehow normative.

HOPE tackles those two false notions by doing a couple of things. One, it measures progress toward equity using measures that are oriented in a positive direction. So, for example, instead of measuring poverty rates, the HOPE investigators measured the share of a population group that has a livable income. The measures are oriented in a positive direction to inspire action, to inspire ideas about what could possibly be done as opposed to the deficit orientation that is too often the case. What the HOPE investigators did was to actually measure which is the healthiest population by race, ethnicity, or socioeconomic factors. It will be no surprise to epidemiologists who are reading this that it is not the White population writ large that is the healthiest population in most instances. It is that population of folks who have the highest socioeconomic status, in this case, as measured by educational attainment or income.

On all of the HOPE's 27 measures, the populations that fared the best were those with the highest levels of education, college degrees or higher, or the highest levels of income, in most cases, at 400% of the poverty level or higher. In so doing, it compares all racial, ethnic, and socioeconomic groups against the population that is faring best.

In this way, the goals that HOPE has established are the health status that the healthiest populations have achieved. And, in so doing, HOPE's researchers do not use the White population as the reference group or comparison group. Secondly, HOPE gives policymakers and advocates a sense of which states are doing best, and in so doing, we can begin to identify states that have a longer distance to achieve the goal and those that have a shorter distance to achieve the goal.

We might be able to find patterns in terms of policy strategies that those states are doing that are closest to achieving the HOPE goals and to see if those policy strategies can be replicated in other states as well. HOPE represents a new way of measuring progress toward equity, one that is aspirational and, at the same time, can begin to point to specific concrete policy actions that can be taken by policymakers and leaders who have the goal of working toward creating the healthiest populations from every racial and ethnic group in their state and in the nation as well.

***Dr. Christopher:* Thank you, Brian, and we want to make sure people know that they can access that interactive database. The interactive HOPE Database is still available for organizers, activists, local community leaders, state-level legislators to use as a tool for establishing very clear, measurable goals and holding themselves accountable for achieving those goals. Thank you for your leadership in creating that database and for sharing with us the insights about it.**

**Clearly, our elected and appointed officials have a critical role to play in helping our nation move forward in this long journey of overcoming our legacy of racism, racial hierarchy, and the actual codified belief in a false hierarchy of human value. Attorney General Herring, we know you took a courageous step in reversing decisions that have been made by your predecessors, and you did so not so long ago in an era where there was actual denial about the truth of racism. We have moved into a moment right now in our country where people are denying that racism exists, and your decision to reverse some of the opinions of your predecessors was profound and has had a meaningful impact. Mark Herring, would you share with us why you felt it was so important to formally reverse the opinions of your predecessors that were rooted in racial hierarchy?**

**Attorney General Mark Herring:** We in Virginia often talk about having over 400 years of history since the founding of Jamestown. When you do that, sometimes, it is easy or perhaps convenient to think that all these bad things happened way back then, so we don't need to talk about them now. But one thing our work showed was that these opinions date from reconstruction to well into the latter half of the 20th century, which wasn't really that long ago.

The opinions are based on laws, however, that have been repealed or superseded by federal law or judicial opinions, so your question is a fair one. Why do this? What does it matter? Well, I am here to tell you it mattered a lot. It was important to do. It was an important step toward true reconciliation, and it is work that I think state attorneys general have a unique responsibility to do because of their place in the legal system.

The work we did shed important light on the history of racism in Virginia. The attorney general opinions we unearthed demonstrated that attorneys general were far from passive observers of Jim Crow laws, segregation, and massive resistance. The words of attorneys general carried weight and influence in their time, just as the words of our current attorneys general carry weight and influence today. Some of the opinions even showed that some of the attorneys general were champions for racist laws. These opinions covered an expansive range of laws, from poll taxes and other racially restrictive barriers to voting, bans on marriage, and segregation in schools, transportation, and other public spaces.

So, the work documents a significant part of the role the state's attorneys general, in particular, and the legal system more broadly, had in cementing and perpetuating the long history of racial injustice in Virginia that disenfranchised Black citizens and blocked their political and social advancement. In doing so, it also helps Virginians of today to understand how the impact of racial injustice toward Black citizens can persist well beyond the civil rights movement and well beyond the development of the Equal Protection doctrine. Further, it encourages Virginians of today to intentionally look around and identify where racial inequity persists in structures and institutions of government and society and then to do something about them, to set about rectifying them.

We found that Virginia's attorneys general had done nothing to renounce and reject those racist opinions of the past. It is never too late to right the wrongs of the past. By finally doing so, we could take another step toward closing the chapters of racist laws in Virginia and in our history and show they are not a reflection of the Virginia of today nor what we want for the future of our Commonwealth.

***Dr. Christopher:* Thank you, Mark Herring. That is such an important story, and I am sure it gave rise to other stories. It is an important body of work, and we are very happy to have an opportunity to hear about it. It is amazing how individuals can make a difference. We have heard from Brian Smedley, Mark Herring, two leaders, two individuals that made decisions that mattered, that made a difference. Some of us remember that during the Clinton administration, there was presidential leadership in the launch of a Clinton Commission on Race. Few of us know the backstory in terms of how that came about. We are finding that presidential leadership in the history of this country has often been the critical factor in progress in terms of issues of race and racism. Mike Wenger has been a leader and an advocate in this space for a very long time and has a unique story to tell about his role in that Clinton Commission on Race.**

**Mike, can you briefly tell us the story of how you influenced President Clinton's decision to create a Commission on Race in America during his presidency? Why is it important that there be presidential leadership on this vital issue today?**

**Mr. Mike Wenger:** In November 1996, shortly after Bill Clinton was re-elected, I was giving a speech at Hilton Head in my role as the representative of the governors of the 13 Appalachian states at the Appalachian Regional Commission. In the speech, I talked a lot about the importance of a community's meaningful racial diversity in attracting and sustaining economic development.

As my wife and I were driving up I-95 back to Maryland, I was complaining that President Clinton, despite his verbal commitment to racial justice, had done relatively little during his first term to change the racial climate in the country, and I was fearful that his second term would be more of the same. My wife listened for a while and then said something to the effect that I should stop whining and do something about it. That led to a discussion of public-private partnerships, which led to a discussion of the President's Council on Physical Fitness, with which she had become familiar when she was in high school in the 1960s. It had had a significant impact on her life and had changed the national climate regarding the importance of healthy living—don't smoke, exercise regularly, et cetera. Those of us old enough to remember will recognize the dramatic changes in the past 60-plus years in reducing smoking and in the explosion of physical fitness centers across the country.

Could we, in fact, have a similar impact on the racial climate in this country? So, when we got home, I wrote a brief concept paper advocating for the creation of a president's council on racial reconciliation, and through friendships I had developed with then Mississippi Governor William Winter and Secretary of Transportation Rodney Slater, the paper made its way to President Clinton's desk. And while the concept changed significantly in the planning stages, it did form the basis for what became President Clinton's initiative on race.

I had the honor of being a deputy director of the initiative, and I learned that despite the challenges of political considerations in virtually every decision we made, Presidential leadership was essential in gaining the public's attention, giving the effort credibility, engaging new allies, and getting your calls returned. Political considerations, like budget limitations, political positioning, unanticipated intervening events, did limit our accomplishments. But the initiative energized and engaged people who cared about racism and has led over the years to connections that would not have otherwise occurred. Many people who, years later, got involved in the TRHT movement were energized by the Clinton initiative. For example, one of the leading organizations in the TRHT movement is the American Association of Colleges and Universities with which we had worked during the Clinton initiative. They now have TRHT Campus Centers on more than 70 college campuses.

So, three lessons that I learned from that experience—one, listen to your spouse. Second, you cannot judge the effectiveness of any long-term effort by the immediate results but rather by the long-term impact it can have. And, while any effort to address racism must be as free as possible from political influence, Presidential leadership is absolutely indispensable.

***Dr. Christopher:* Thank you, Mike Wenger, for sharing that story. I have heard it before, but I really wanted others to hear it and to be reminded that it is a marathon, it is not a sprint.**

**As I talked about the TRHT framework, we have heard about narrative change being a critical pillar, and all of this work has helped to change the narrative. The Clinton commission worked with colleges and with local communities, which helped to foster healing, racial healing. But we know that racism has been embedded into our systems and structures, and we asked ourselves when we were creating TRHT how has this persisted for all these centuries. And we answered the question by coming up with three primary vehicles or avenues for maintaining racial hierarchy, and the first was separation, as I said earlier.**

**The second was the legal system, and, finally, our economy. But separation took many, many forms throughout history and still, today, includes residential racial segregation and the residual effects of relining, reservations to which Native American communities were confined, even our immigration policies. We need to really look at the vehicles by which racism is sustained so that we can come up with interventions to reverse them.**

**Dr. Eaton, you have written eloquently. About the ways in which racism has created separation within American society. Can you give us specific examples of how systems like education and housing reflect the racial hierarchy, ideology, and practice?**

**Dr. Susan Eaton:** In the past couple of decades we've learned so much about how the separation and inequalities we see in contemporary society are direct outgrowths of racial hierarchy ideology in the distant past, the recent past and today.

If you were to overlay a redlining map from the 1930s with existing metro areas and cities and we see that the places our own government deemed “inferior” or “bad investment” because of residents' phenotype are still today some of the most unequal most segregated regions in our very unequal nation.

Racial segregation—an outgrowth of racial hierarchy ideology, concentrates poverty in particular neighborhoods and schools. This condition compounds the harms of individual poverty.

The harms of concentrated poverty—more exposure to pollution, less access to health care, healthy food and to adequately resourced public schools, more exposure to violence, incarceration, poor quality housing—are not shared equally. Nationally, about 24% of Native Americans, 20% of Black Americans and 17% of Latinos live in high poverty neighborhoods compared to just 4% of white residents.

It is even more extreme in our schools with 74% of Black and Latino, 70% of Native American and just 32% of white students attending schools where at least half of the students qualify for free and reduced lunch.

Several investigations in recent years have found that the highest exposures to unhealthy, often life-threatening levels of heat follow the path of historic and current day segregation. Recent studies demonstrate that residential segregation exacerbated racial disparities in access to testing for COVID-19, infection and even death for Black residents. Testing sites were more common in white neighborhoods as was access to care.

Tufts University Professor Daanika Gordon's in-depth study of a segregated Rust Belt region, details the mechanisms of both over-surveillance of a Black neighborhood, which is also underserved by emergency services. She found the opposite in the nearby predominantly white neighborhood.

Schools that serve large shares of students in poverty also tend to be under-resourced. Educators there must tend to an array of social challenges, such as hunger, homelessness and the economic precarity of families. It is more difficult to attract and retain high-quality teachers to higher poverty schools. Such schools tend to have higher rates of teacher absenteeism and less experienced teachers. Such schools tend to have fewer opportunities for advanced coursework and are typically less likely to have counselors or nurses on permanent staff and more likely to have security guards. A 2017 investigation by the non-profit EdBuild, found that despite educating the same number of students, school districts with larger shares of Black and Latino students receive $23 billion less overall than predominantly white schools.

One way that segregation engenders these disparities is because racially identifiable areas and institutions make it easier for government officials, private investors and businesses, to overlook communities of color and focus instead on serving the interests of the equally easily identified, more powerful, wealthier, whiter communities. Similarly, easily identifiable Black and Latino neighborhoods make it easier for authorities to over-surveille and punish them. Research even indicates that segregation enabled predatory targeting by the real estate finance industry.

***Dr. Christopher:* Thank you, Susan Eaton. Your work is so powerful and has been for many, many decades. As we have talked about so far today, we know that the messages that are being conveyed, that are being heard and ultimately internalized and acted upon by people across this country determine the temperature and the pulse and influence our willingness to move forward with real transformational change. Sometimes, it is a major book or publication. I remember back in the ’70s, the book *Roots* and certainly the accompanying television series opened a level of awareness. It awakened so many people to the very idea of slavery in America and the felt sense of its impact on human beings. Books come along that really do change our way of thinking and seeing and looking—and so our next speaker is bestselling author Heather McGhee.**

**Heather McGhee, your award-winning book, *The Sum of Us—What Racism Costs Everyone and How We Can Prosper Together*, offers readers a new way of thinking about the benefits of overcoming our divisions rooted in racial hierarchy. Can you contextualize this zero-sum mentality and how it has given rise to what you call “drained-pool policies”?**

**Ms. Heather Mcghee:** Thank you so much, Dr. Christopher. I spent nearly 20 years working in domestic economic policy, mostly at a think tank called Demos, which was focused on finding solutions to inequality in America. Obviously, the area of health care is one of the most unjustly unequal areas of our society, and yet virtually every aspect of a family's life is touched by a set of public policies that have been shaped with indifference over time to the racial and class and gender inequities and often deliberately, usually at their outset, to further these inequities.^†^

And so I set out to write a book that would speak to a very broad audience, perhaps an audience that had never picked up a book about the economy before or picked up a book about racism before but an audience that might have asked themselves the question that is the opening line in my book, which is, “Have you ever wondered why it seems like we can't have nice things in America?”

And by nice things, I do not mean flying cars. I mean truly universal health insurance and paid family leave and a well-funded public school in every neighborhood. These are the kinds of things that a country with our wealth and riches should be able to afford for her people, and yet they are out of reach of so many of us. And what I realized and learned over the course of the journey that it took me to write *The Sum of Us* is that racism in our politics and our policy making is so pervasive that it leaves no system that shapes our lives untouched, and in so doing, it creates a cost for everyone. One of the main vehicles for this racist thinking in our politics and our policy making is a zero-sum mindset. It is a story, a story often sold and maintained and broadcast by people with a lot of wealth and power who are very satisfied with the status quo, that tells many Americans to resent one another, tells particularly white Americans that progress for people of color must come at their expense.

It is a lie. Economists know that the economy is not a zero-sum game, that, in fact, inequality costs are growing. The “fringe left-wing think tank” Citigroup calculated that the Black-white economic divide had cost the U.S. GDP $16 trillion over 20 years.^‡^ And yet still this zero-sum narrative that says that there is sort of a fixed pie of well-being, and if one group gets a bigger slice, the other group must get a smaller slice—that is a pervasive mindset.

It ultimately leads to really self-sabotaging effects because what happens is that oftentimes zero-sum thinking leads to fear of loss of status, of resentment toward people who are seen as competitors, and therefore, dynamics happen in society which make groups less willing to have things in common that may make their neighbors better off if they are worried that it could interrupt their sense of status.

We saw that very vividly in a phenomenon that happened across the country in the 1950s and ’60s, well into the 1970s, actually, where there was a Supreme Court case about this, which was when towns and cities were willing to look at their big, beautiful, well-funded public swimming pools that were these glittering public goods. And they were willing, if they were segregated, to destroy them, to literally drain out the water of their public pools rather than submit to federal court mandates for integration of these public pools.^§^

So that idea that white communities would be willing to destroy or, in some cases, privatize their public goods, take something that was free and for the benefit of the public, though on a racially exclusive basis, and then turn it into either a private luxury or destroy it altogether really, for me, began to stand for a broader phenomenon on our politics that explains the reluctance of many Americans to share across race to find common solutions to common problems, because ultimately, we have to all swim in the same pool if we are going to solve so many of our most significant ills.

We need to work together to stop planetary climate change. We must fund public goods together whether it is well-funded public schools or infrastructure or universal health care. We need to be willing to bargain collectively for higher wages and better benefits on the job. And in a multiracial society, it is going to take cross-racial solidarity, empathy, and the rejection of zero-sum thinking in order for us to counter that drained-pool politics, to refill the pool of public goods for the betterment of all.

***Dr. Christopher:* Thank you so much, Heather McGhee. Collective work is required. It is going to take a narrative that helps to move us to that point of working together, and as I said earlier, oftentimes, it is a book like yours and others that can help to shift the collective public way of thinking and way of seeing. There was a book that was published 20 years ago. It was called *Unequal Treatment*, and Brian Smedley played a key role in editing that landmark publication. It helped to move people past denial, I would say, of the reality of inequities within the health care system.**

**Brian, it has been more than 20 years since the release of the landmark publication *Unequal Treatment—Confronting Racial and Ethnic Disparities in Health Care*. Can you summarize how this book shifted the broader narrative and perspective about health inequities? What, if any, progress has resulted over the last 20 years?**

**Dr. Smedley:** Thank you for that great question. It is important to recognize that when you look back to the 1980s, 1990s, when the federal government was just beginning to turn its attention to the problem of racial and ethnic disparities in health status and access to health care, that many researchers, many health care providers, many leaders in the health care industry did not believe that patients of color could receive a lower quality of health care than white patients simply because of their race. This was a controversial question although it is hard to believe now. The *Unequal Treatment* report was important because it documented conclusively that patients of color experience a lower quality of health care than white patients, even when access-related factors such as whether or not you have high-quality health insurance or money to pay for out-of-pocket costs are taken into account, and in so doing, it demonstrated how pervasive racism is.

I suspect that before publication of the *Unequal Treatment* report, most Americans would think that health care would be the sector least infected by racism operating at multiple levels. We can see with our own eyes instances of injustice when police officers abuse and mistreat people of color on our streets. We can see with our own eyes when people of color are sentenced to harsher sentences for the same crimes as white defendants. We expect that kind of racism. But I think most Americans, before the *Unequal Treatment* report, would suspect that health care would be the sector least likely to have evidence of racism in its systems. But the report's careful documentation ensured that the narrative changed, that racism and other systemic factors were clearly implicated behind the lower quality of services that patients of color receive. We are talking about across a range of clinical services from A to Z.

The report also showed how bias, both explicit and nonconscious biases, can affect the thinking and behavior of health care providers and the kinds of contextual factors and settings that tend to exacerbate them. So first, health care providers we tend to think of as being highly trained, highly dedicated, committed to the care and well-being of their patients, and I do not doubt that the vast majority believe in their hearts that that is the case. But *Unequal Treatment*, as well as now decades of research, show that health care providers are human beings like the rest of us, subject to biases that they are aware of as well as biases that they may not be aware of. Researchers have been able to show that providers with higher levels of nonconscious racial bias are less likely to prescribe the most effective treatments and services for patients of color who present with the very same symptoms as White patients.

It also showed that contextual factors are important, and they are important from a policy perspective. Research has shown, for example, that health care providers, regardless of training or intent, are much more likely to rely on biases, on unconscious and conscious biases, under conditions of time constraints, resource constraints, and cognitive complexity. In other words, when a health care provider sees a patient, sometimes, they do not have time to get a full history on that patient to fully understand the patient's social and economic circumstances.

Oftentimes, providers are constrained in terms of the kinds of diagnostic tests they can order, and providers often face high levels of cognitive complexity, particularly when patients present with complex medical conditions. Well, almost all providers will tell you that those very conditions—time constraints, resource constraints, and cognitive complexity—are the very conditions that are likely to elicit biases, both nonconscious and conscious biases among providers. And ironically, these conditions are more likely to be found in the under-resourced health care systems where patients of color are more likely to get their care—such as safety net institutions. Why is that? Because these providers are trying to fill in missing information, but those biases can often be harmful in terms of that provider's ability to diagnose and treat the problems that these patients face.

In terms of progress, yes, there has been some progress. The Affordable Care Act, passed under President Obama, helped to expand insurance coverage in the United States, and it contained many nondiscrimination provisions that are important to help guard against discrimination on the basis of race, ethnicity, sexual orientation, gender identity, language ability, and other protected categories. But the Affordable Care Act, from a racial equity standpoint, largely preserved the status quo of medical care as a market commodity in the United States, and I do not think that we will achieve racial equity in health care as long as health care is a market commodity because with that kind of financial incentive, the healthiest, wealthiest patients will be the ones who fare best in our health care systems, often excluding and leaving fewer resources for those patients burdened with high levels of illness and lacking the resources to afford the best quality health care.

So, I think our story is a mixed one. Yes, there has been some progress, but unfortunately, we have been unable to significantly reduce the health care quality and access gap between White patients and patients of color. That is due to many complex factors, bias among them, but I think as the example that I provided about the conditions in which biases are most likely to be elicited shows, policy strategies are important to help address these challenges.

***Dr. Christopher:* Thank you so much, Brian. If we look at this from the decision to shut down public good like swimming pools to the reality of inequities in treatment in the health care system, we see very tangibly how this belief system in a false taxonomy, a hierarchy of humanity, of human value pervades our society and pervades our democracy. And, hopefully, we feel the urgency to put it behind us. It is a relic of many centuries past, and we need to overcome it. That is why I was so impressed with the foresight, insight, and courage of Attorney General Mark Herring because he recognized that looking back is a necessary step for moving forward. That is part of the truth. That is part of the transformation. Although you were in Virginia, the capital of the Confederacy at one point, and you did that work there, I would like to think that your work is influencing others today, other attorneys general, and I am wondering if you have any advice, Mark, for your peers.**

**What advice would you give to your peers and other leaders in states across America about undertaking this important work?**

**Attorney General Herring:** Well, first of all, I have learned to be very careful about giving advice to other public officials, but what I would do is offer encouragement and share my experiences which were all at the state and local level, but experiences that they can nonetheless draw from.

One thing I learned is you can do this work in a way that promotes unity. I found that we in Virginia—and I am convinced the same holds true across the nation—are strong enough that we can talk about hard issues, including those that involve race. I also found that we are all better off when we talk about history honestly and that when you really engage with people, they feel that way, too. I will share a couple of examples.

My team and I successfully defended the removal of the enormous statue of Robert E. Lee from Monument Avenue in Richmond. We made much of our case by introducing a mountain of evidence that this statue was erected by the white power structure in 1890 as a tool to reassert themselves and as part of a deliberate and intentional effort to intimidate and degrade Black Virginians and suppress the growing civil rights movement. The case ended with a unanimous Virginia Supreme Court agreeing with us and with the removal of the statue as part of a deliberate and intentional effort to heal and move us forward together.

To be sure, the conversations we had as a commonwealth during that 1-year legal fight were sometimes hard and emotional. Many white Virginians finally confronted the safety and privilege our skin color afforded us, something Black Virginians did not have the luxury of feeling. I will never forget seeing the impact the removal of the statue had for many Black Virginians, including many who had lived in Richmond all of their lives and had wondered, “Would this day ever come?” It seemed like a heavy weight was being lifted from their shoulders.

I would also share with public officials that sometimes you are not even aware of the ways in which this work can make people's lives better at the time you set out to do it. Going back to the overruling of prior attorney general opinions that were rooted in a false hierarchy…I heard from so many people how much it meant to them, especially those who had lived through it or remembered stories from their parents who lived through it, to have an attorney general acknowledge the role attorneys general had—and the role the legal system had—in perpetuating racism and injustice and formally renouncing those opinions. It, too, seemed like another weight lifted from their shoulders.

We also found that you do not really need to wait around for some unique moment or some organized movement to get you started. We found what was most important was making the commitment to get to work and to take action, and once you do that, the rest falls into place. Yes, there is planning, organizing, everything else that goes into changing laws and systems and public policy, but you will find that when you do that work in a way that promotes unity, there will be support for you in the communities you serve.

***Dr. Christopher:* That is what our survey showed, that there is a felt need for this coming together to solve our collective challenges, and I appreciate hearing about that work in the state of Virginia. There is a movement right now in this country that has gained a lot of steam, recently, which is this movement for actual reparations or payment or compensation for the centuries of unpaid labor by African Americans. In fact, this very week, over a hundred local and state leaders gathered in Evanston, Illinois, to begin to have more of a sense of community in terms of state and local efforts at reparations or repair as part of the healing process.**

**Mike Wenger, you've written about the issue of transformational reparations. Can you tell us what a transformational strategy would look like and where racial healing fits in this strategy?**

**Mr. Wenger:** My colleague Rob Corcoran and I have written a paper for the NCHE entitled “Toward Transformative Reparations.” We argue that monetary compensation alone, without meaningful accompanying efforts to promote racial healing, is woefully inadequate to atone for our past and current sins, including our oppression of Indigenous people, and, in fact, would actually create deeper racial divisions without addressing the deep-seated racism that persists in our society.

We believe that monetary compensation without healing will not eliminate the structural racism that exists in virtually every institution of our society. It will create further divisions and resentments by ignoring economic injustices that other population groups confront. It will be seen by people in power as relieving them of any further responsibility for eliminating inequities in our society. You can just hear them saying, “Well, we've done this. What more can they want?” It will make it impossible to obtain the resources necessary for meaningful monetary reparations, and it will further weaken our democracy by maintaining our divisions and therefore making it more difficult to confront the growing authoritarianism in our society.

In looking specifically at recent Australian and Canadian experiences as well as our personal experiences, especially Rob's transformative trust-building work in Richmond, Virginia, over a period of more than 30 years, my work with the Clinton initiative, as well as the work of both of us with TRHT, we have developed a set of principles that communities can adapt to their unique circumstances in promoting racial healing along with reparations efforts in their communities.

First, there must be an accurate recounting of our history, both local and national. Sadly, the controversies over book banning and school curricula are taking us in the opposite direction. Second, a critical mass of diverse community members must embrace a common vision of our future and share goals that will build momentum to achieve that vision. Third, we need to understand and respect the different cultures, experiences, and perspectives that coexist in our communities. Fourth, we all have stories to share, and we must share them honestly while listening deeply and without judgment to the stories of others and avoiding blaming and shaming. A colleague, Liz Medicine Crow, often says that the shortest distance between two people is a story. Fifth, we must commit to both restorative justice and changes in institutional policies and practices. Sixth, key institutions and their leaders as well as grassroots organizations must be engaged.

Seventh, one very clear lesson from the Australian and Canadian experiences is that political leaders from across the spectrum of ideologies need to be involved. Eighth, transparent communications are essential to building support and overcoming opposition. Ninth, adequate resources are essential, especially from the community and the private sector both to launch this effort and to sustain it, and finally, we must create teachable moments from the tensions that inevitably arise.

So, with these principles in mind, we have recommended the following next steps. First, incorporate the healing strategies of trust-building and TRHT into the charge of the commission on reparations proposed in HR 40 and include our Indigenous population. Second, engage elected officials at every level of government working through organizations like the National Governors Association, the Council of State Governments, National League of Cities, National Association of County Officials, and others. Third, engage nonprofit and faith organizations. Some examples are: Everyday Democracy, Community Action Partnership, American Library Association, American Association of Colleges and Universities. Fourth, ask the Biden administration to create by executive order a bipartisan and diverse commission on transformative reparations. Five, launch a major public education campaign to raise public awareness about the importance of this effort, and finally, pursue both government and private sector resources, especially from corporate and philanthropic interests whose history includes profit-making from the oppression of others.

***Dr. Christopher:* Thank you, Mike. It is clear that you have devoted a great deal of thought and commitment to this work over many decades, and we are honored to have you as part of this conversation today. Clearly, you are calling for a comprehensive approach to this work, and that is embedded and embodied in the TRHT strategy. There are many parallel movements right now in this country that give us a lot of hope, and these movements are not polarizing in their depth. People may be exploiting them for the purposes of gain and division, but these movements are definitely moving America forward to help with overcoming our legacy of racism. There is the redress movement, which is looking at the consequences of housing segregation and separation in this country, and there are some exciting things happening to mitigate and redress that.**

**Dr. Eaton, can you give us examples of innovative policies and strategies that are being put in place to redress and mitigate this nation's legacy of separation?**

**Dr. Eaton:** Yes, there are a lot of exciting things happening. I'll quickly summarize a few with the hope that folks will want to learn more on their own.

Two different types of strategies seem to have emerged.

In the first category are efforts to reduce or prevent worsening segregation. This often includes new policies and practices to reduce segregation perhaps through redrawing school district boundaries or writing inclusive zoning regulations.

In the second category are efforts that don't necessarily reduce segregation per se, but account for and repair the damage segregation has caused in various areas of life, through, say, broad investments in communities of color or the provision of particular services such as parks or health care facilities.

Both of these approaches are crucial.

1. In that first category, are the 30 or so communities, regions and states, including, Long Island, N.Y., Richmond, Virginia, and Columbus, Ohio that participate in housing mobility programs. These programs enable low-income families with children to use federally funded and locally administered Vouchers to move to neighborhoods with less poverty, higher resourced schools, less crime, and more resources such as grocery stores and parks. These programs typically offer a range of services, including assistance searching for housing, and short-term financial help.

Also in that category sits The Century Foundation's Bridges Collaborative, a hub for educators and other practitioners working to create and sustain racially and socioeconomically integrated schools and neighborhoods. The member organization provides space for local actors to “learn from one another, build grassroots political support, and develop successful strategies for integration.”

In the category of redress, there is also a lot of really great stuff happening and again, I recommend checking out the non-profit organization, Redress Movement, to learn more.

Using funds earned from the sales of marijuana, the Chicago suburb, Evanston, Illinois approved its Restorative Housing Program in 2020, which is the city's first reparations initiative. Acknowledging “the harm caused to African-Americans” from discriminatory housing policy and practices and inaction on the part of the City from 1919 to 1969,” in its first year, the program provided 16 African American residents $25,000 each to put toward a down payment on a home, mortgage payments or home repairs.

After advocacy and activism and community-based education around the history and harms of segregation in the region, in 2021, officials in the city of St. Paul, Minnesota's capital, apologized for the municipality's role in discrimination against African Americans. They acknowledged that the practices and inaction on the part of the city contributed to long-term harms, particularly in housing and wealth. Part of the remedy is the Inheritance Fund, provides housing-related assistance to descendants of residents of the predominantly Black Rondo neighborhood in St. Paul, who were displaced and then harmed by policies that created and exacerbated segregation.

I highly recommend the book Just Action by Leah and Richard Rothstein. This book details policy, practice and movement solutions related to housing segregation. But we know of course, housing policy is education policy and vice versa.

***Dr. Christopher:* I so appreciate your commitment to this work, Susan, and the publications and the resources that you continue to make available to local leaders and to researchers. Again, the work of communication is foundational to the transformation that we all seek. And Heather McGhee, I know your book is having a tremendous impact, and you have been traveling around the country communicating to audiences of sometimes thousands of people. You also translated your book into a podcast that was supported by the Obamas' communications company, a podcast of the same name, *The Sum of Us*.**

**In your journeys to research the book and most recently to develop the podcast of the same title, you encountered many cross-racial coalitions that were driving successful change. Can you give us an example of these local coalitions?**

**Ms. McGhee:** One of the most exciting ideas that I discovered on the journey to write *The Sum of Us* was the concept that there can be real gains when people come together across lines of race to create multiracial coalitions that require multiracial solidarity and therefore are able to activate something of a dividend, a gain, some outcome that is really attributable to the kinds of organizing and collective action that simply cannot happen when we succumb to divide-and-conquer, us-versus-them politics. I set out to find as many stories as I could tell about solidarity dividends. There are some in the book and then the podcast series, the documentary series highlighting eight original stories of solidarity dividends in places from Maine to Memphis to rural Nevada.

Because there has been a recent sort of coda to one of the stories, I think I would like to tell the story of what happened when a very unlikely pair, a white extreme sports mountain biker, surfer, mountain climber found out that the town near where he likes to do outdoor adventure had a siren, an air raid siren that would go off at around sundown every day, and that it had originally been erected, this siren in the middle of this small rural town called Minden, Nevada, to be a sundown siren to tell the Indigenous people, the original inhabitants of that land, that they had to get out of town or face violence and arrest. And that sundown siren was still blaring in 2020 when this story of this unlikely pairing began. And so Matt, white guy, adventure sports guy, found a local Washoe retired educator, Marty Meedan, who had made it his life's work to educate Indigenous children and to educate communities about Indigenous history, and the two of them teamed up to try to bring more attention to what had, of course, for the local Indigenous community been this stain, this blight, this triggering, traumatic daily occurrence of this sundown siren.

The local white sheriff, town leaders, local folks who walked down the Main Street had various excuses for why it was not a problem that there was still a sundown siren. Many of them acknowledged the fact that it was originally a sundown siren, but they said, “Well, we don't have that law anymore.” And, “That's not what it means anymore,” and: “Now, it's just a nice tradition.” “It tells me it's time to get home.” “It tells me what time it is.” “There's another one that happens in the middle of the day, so that's like a lunch bell.” “We just like it.”

It reminds me so much of the work that Attorney General Herring did in Virginia to take down these monuments and why it is so important and how it is that people today, usually white people, are able to create an alternative narrative that imbues these violent, racist symbols with a benign emotional meaning. That, of course, it sets up this us-versus-them dynamic where something that is a source of pride for white residents is a source of trauma for residents of color, and there is no communication across that barrier. So, here come Marty and Matt, who are trying to create in this town of hundreds of people, surrounded by open land and by a number of reservation communities, a sense of momentum that this has to change. Ultimately, they were able to do so by creating events that brought people together, including in partnership with a young Indigenous Washoe Paiute runner, a teenager who was one of the fastest distance runners not only in his state but in the country.

Distance running is a traditional Indigenous sport that is really beloved in Indigenous communities today, and for this young man, who ran and was one of the fastest runners in the country, he really did so in honor of his great-grandfather, who ran away three times from a Native boarding school that had kidnapped him as a child as part of the long and horrific legacy of Indian boarding schools. So as part of a way to bring more people's attention to the siren, to the importance of this anti-Native history, they created a run and a mountain bike ride that would go across the path that the great-grandfather took from the old boarding school back to his homelands, and they would do it at the time of the siren. And they would use it to raise money for a campaign to silence the siren.

Ultimately, just recently, this year, the siren finally sounded its last, and there was celebration and tears and a sense that even in a town where people love their history, love their markers and their plaques—you cannot go 50 feet without a plaque about how this building used to be one of the first pharmacies, and this used to be a cobbler. But there is no plaque where the siren was. You have this potential for often new people who are not from the community to come in and give a basis for a cross-racial, cross-cultural coalition that says that we can all benefit from turning the page against our racist history and moving forward together.

***Dr. Christopher:* Wow. Thank you so much for that story. I can hear the siren in my head as you told it. I cannot imagine what impact that had psychologically on the people of the community who trace their ancestry back to its intentional harms and terror. It is comforting to know that since the podcast aired, there has been a resolution and today it is a story of success and healing. We are fortunate to have you share that with us. Thank you so much, Heather.**

**We have heard such important information from all of you on this roundtable today, and it all reminds us that there is, in fact, not only a readiness but, indeed, a hunger for a way forward to solve our nation's need to overcome its legacy of racial hierarchy and division and move forward together. I am inviting you to give us some final thoughts.**

**Can each of you tell us why this work of overcoming our legacy of division rooted in racial hierarchy is vital to the health of our current democracy? Brian, would you answer this question as it relates to health equity? Attorney General Herring, can you answer as it refers to our nation's legal and justice challenges? Mike, can you answer from the standpoint of your call for transformational reparations? Susan, can you answer from the perspective of opportunity for future generations? And Heather, would you answer from the perspective of our collective benefits and subsequent democratic viability?**

**Dr. Smedley:** Thank you for that question. I would say that health inequities, like wealth inequity, income inequity, social and political inequity, are absolutely corrosive to democracy. Health inequity, first of all, means that America is less healthy. People of color are part of what constitutes America's health, and so to the extent that we are seeing a heavy burden of avoidable disease, disability, and premature death, health inequity means that America is less healthy. It means that we pay more for health care. It means that we are losing valued members of our communities way too early. People who are less healthy are often less able to participate in our democracy and make their voices heard. But I also think that the persistence of inequity also relates to the question that Heather posed at the outset.

Why can't we have nice things? One of those nice things, as she noted, is a universal health care system that would address everyone's needs. Another influential book is Jonathan Metzl's book *Dying of Whiteness*, where Jonathan talks to individuals, Whites, who say that a universal health plan, while appealing and while it may provide some benefits to them, is not something that they could politically support because they do not want to see, quote, “undeserving people of color” receive that benefit.

This is one example of a pervasive strand of racism that is built into our narratives, built into our understanding of policy, that we want to establish policy for some people who are perceived as “deserving,” and in the American mind, that is often the White population. Health inequity not only makes us less healthy as a nation, and inequity broadly impedes our ability to find solutions that would benefit all of us.

**Attorney General Herring:** I would say as a preliminary matter, at least to me, there is an element of this work that is a moral imperative. I fundamentally believe that we are all people, and everyone deserves to be treated equally, fairly, and with dignity. So, to me, there is a moral component to this work, but overcoming our legacy of division rooted in racial hierarchy is also vital to meeting the challenges our legal and justice systems face. These systems are integrally tied to our democracy and some of our most fundamental beliefs, even our national identity as Americans.

If you ask most Americans if they thought it was vital to the health of our democracy to have a justice system that was fair and treated all people equally, regardless of their race or economic status, I suspect there would be wide agreement. To paraphrase Heather McGhee, that would be a nice thing to have for all of us, wouldn't it—a legal and justice system that treated everyone fairly and equally?

When I was attorney general, I saw firsthand how the weight of our system of criminalizing possession of small amounts of marijuana fell disproportionately on Black Virginians, which compelled me to call for change, and we succeeded in ending it. The same was true for our system of cash bail, training for fair and impartial policing, and so much more.

But how quickly we have seen backsliding and calls for undoing the progress toward a more fair, more just legal and judicial system and—parenthetically, I would add—safer communities. Those calls are made in race-neutral terms, of course, but often, the old stereotypes and the us-versus-them framing are clearly recognizable. I have not given up my belief in working to change laws and policies to help make peoples' lives better. Far from it. But sometimes, when laws change for the better but the beliefs that underpin the old unjust laws and unfair systems do not change, that progress can be fleeting.

***Dr. Christopher:* Thank you so much, Attorney General Herring. And that is why the work of addressing the fundamental belief system is such an imperative. Mike?**

**Mr. Wenger:** I believe that in my lifetime, the danger of losing our democracy has never been greater. If we do not narrow our political and racial divisions, we will simply not have the strength to confront the existing authoritarian threat. I take heart from a meeting on July 18, 2022, between a delegation of global reparations leaders and representatives of Pope Francis to discuss the Catholic Church's role in supporting and benefiting from the transatlantic slave trade and its legacy. The delegation called on the pope, “to urgently lead the world in building a global culture of repair and healing and otherwise taking transformative action for Africa and its worldwide diaspora.” One week later, Pope Francis issued an historic apology for the ways in which the church cooperated in Canada's catastrophic policy of Indigenous residential schools, a policy which we were guilty of in this country, and he implored God's “forgiveness for the sins of her children.”

It will require faith, courage, and perseverance, but we can do this. We also might take heart from the words of the late former Illinois Senator Paul Simon, who, in 1988, ran unsuccessfully for the Democratic Party presidential nomination and asserted, quote, “there really is a yearning across this good land for leadership that appeals to the noble in us rather than to the greed in us.” I believe that is still true. In today's toxic political environment, Senator Simon's words may seem naive, but our choice today is whether to capitulate to today's tensions or to be inspired by the response of Pope Francis and rise to the challenge of making Senator Simon prophetic. We think the choice is clear.

***Dr. Christopher:* Thank you, Mike. We certainly agree with you at the NCHE.**

**Susan, your life's work has been to make life better for children, to create a future in which all of our children can thrive.**

**Dr. Eaton:** I think that to have a functioning democracy, it's crucial for young people from different backgrounds to understand and see all the ways in which their fates are linked and break out the kind of zero-sum nonsense that dominates our politics and much of our public conversation.

We know from research in social psychology that the best way to break down racial stereotypes and racism, which are at odds with real democracy, is via cross-racial, cross-ethnic, cross-class friendships. From the work of the economist Raj Chetty and his team, we know that developing even “loose ties” with people in what he terms “connected” communities, with resources and social connections to opportunities, is an important vehicle out of poverty.

We need to create the conditions for those friendships and for young people to lead and do this vital work. The best conditions for this to happen, it seems to me, would be schools and neighborhoods and community settings where young people from different backgrounds can learn and live together under adult mentorship. Segregation has reduced the share of institutions like that, but there are still many out there where educators and students are working to create a healthier democracy.

***Dr. Christopher:* Thank you, Susan. I really appreciate your ongoing work and your brilliant paper that demonstrates how our systems of separation historically and in a contemporary sense can and must be eliminated for the benefit of our nation's children. Heather?**

**Ms. McGhee:** The Heart of America Survey truly shows that a counternarrative exists to the dominant media attention on our political divisions. Yes, the Heart of America Survey says that four out of five Americans believe that the country is more divided now than ever before, but in that same survey, more than four out of five Americans are united on some of the most hot-button issues around race and our democracy, where there have been shocking decisions by state legislatures and by this conservative Supreme Court in the area of diversity policies and in the areas of book bannings and censorship around our children's freedoms to learn.

We have an opportunity, those of us who are focused on sound public policy, on health equity, on outcomes for families and children and communities, to take a high road that does not discount the progress that was made in this unprecedented last decade of movement building among young people, among the immigrant community, among racial justice advocates, the plurality of whom were white people who showed up in majority-white communities across this nation in the summer of 2020 in support of the movement for Black lives, the movement for women's rights and for reproductive freedom.

These movements speak to an enduring and, in fact, growing understanding that our freedoms as Americans are secured by collective action, by people standing up for one another, by the kinds of forward progress that we can never take for granted, and there are demonstrable quantifiable health outcomes for each of those movements' successes. These questions of who we are and who we are to one another and how responsive our democracy is to the will of the people and to protecting our hard-won freedoms is, in so many ways, a question of health as well as one of life, liberty, and the pursuit of happiness.

## Expert Panel

**Dr. Gail Christopher** is an award winning social change agent with expertise in the social determinants of health and well-being and in related public policies. She is known for her pioneering work to infuse holistic health and diversity concepts into public sector programs and policy discourse. Dr. Christopher recently retired from her role as Senior Advisor and Vice President at the W.K. Kellogg Foundation, where she was the driving force behind the America Healing initiative and the Truth, Racial Healing and Transformation effort. Dr. Christopher also served as Kellogg's Vice President for Program Strategy and worked on place-based programming in New Orleans and New Mexico. In 1996 she was elected as a fellow of the National Academy of Public Administration. In 2015 she received the Terrance Keenan Award from Grantmakers in Health. She chaired the Board of the Trust for America's Health from 2012-2022. In 2019, she became a Senior Scholar with George Mason University's Center for the Advancement of Well-Being. Dr. Gail Christopher also became the Executive Director of the National Collaborative for Health Equity in 2019. In 2021, Dr. Christopher was elected by the APHA Governing Council to serve as the APHA Honorary Vice President for the United States.

**Dr. Susan Eaton** is Professor of Practice at Brandeis University's Heller School for Social Policy, where she also directs the Sillerman Center for the Advancement of Social Justice Philanthropy. She has written several books and numerous scholarly and popular articles about the causes of and potential cures and reparation for racial segregation. Her book, *The Children in Room E4: American Education on Trial* (Algonquin, 2008) tells the parallel story of a contemporary civil rights case and a classroom in Hartford, Connecticut. As research director at the Charles Hamilton Houston Institute at Harvard Law School, she co-directed the storytelling project, One Nation Indivisible, which explored efforts at true integration across the nation. In 2021, her book, *The Other Boston Busing Story: What's Won and Lost Across the Boundary Line* (Yale, 2001), about the nation's longest running voluntary school desegregation program, METCO, in Boston, was updated and re-released.

**Mark Herring** served as Virginia's 47th attorney general. During his 8 years as the state's chief legal officer, Mark was at the forefront of high-profile cases and public policy issues confronting Virginians and the nation, including securing the right of same-sex couples to marry, successfully defending the Affordable Care Act, winning the nation's first preliminary injunction blocking a travel ban on Muslims, and successfully defending Virginia's removal of the statue of Robert E. Lee from Monument Avenue in Richmond. Mark also previously served as a member of the Virginia Senate and a member of the Loudoun County Board of Supervisors. He is a currently a partner at Akin Gump Strauss Hauer & Feld LLP, where he co-leads the firm's nationwide state attorneys general practice, and a member of the adjunct faculty at the University of Virginia School of Law where he lectures on the work of state attorneys general.

**Heather McGhee** designs and promotes solutions to inequality in America. Over her career in public policy, Heather has crafted legislation, testified before Congress and helped shape presidential campaign platforms. Her book *The Sum of Us: What Racism Costs Everyone and How We Can Prosper Together* spent 10 weeks on the *New York Times* bestseller list and was longlisted for the National Book Award and Carnegie Medal for Excellence in Nonfiction. The book was also adapted into a Spotify podcast by Higher Ground, the production company of Barack and Michelle Obama and into a young adult readers' version that was released by Random House Children's in February 2023. An influential voice in the media and a former NBC contributor, Heather regularly appears on NBC's *Meet the Press* and MSNBC's *Morning Joe, Deadline White House* and *All In.* She has shared her opinions, writing and research in numerous outlets, including the *Washington Post, New York Times, Wall Street Journal, USA Today, Politico* and *National Public Radio*. For nearly two decades, Heather helped build the non-partisan think tank Demos, serving 4 years as president. She holds a B.A. in American Studies from Yale University and a J.D. from the University of California at Berkeley School of Law. Heather is the chair of the board of Color Of Change, the nation's largest online racial justice organization, and also serves on the boards of the Rockefeller Brothers Fund, the Open Society Foundations' U.S. Programs and Demos.

**Dr. Brian Smedley** is a Senior Fellow at the Urban Institute in Washington, DC where he conducts health equity research and policy analysis, with a focus on addressing structural forms of racism that create and sustain health inequities. Previously, he has held leadership positions at the American Psychological Association (APA), the National Collaborative for Health Equity, and the National Academy of Medicine (formerly Institute of Medicine), where he served as study director for the landmark report Unequal Treatment. His most recent book, with co-authors Drs. Louis Penner, John Dovidio, and Nao Hagiwara, Unequal Health: Anti-Black Racism and the Threat to America's Health, explores the toll of racism on U.S. health and offers policy strategies to mitigate its effects. Dr. Smedley has been honored by the APA, the American Public Health Association, the National Academies (staff award), the Congressional Black Caucus, and the Rainbow/PUSH Coalition, among others.

**Michael R. Wenger** is a Senior Fellow with the American Association of Colleges & Universities. In 1997 and 1998, Mr. Wenger served as Deputy Director for Outreach and Program Development for President Clinton's Initiative on Race. Subsequently, he worked on public policies to address racism at the Joint Center for Political and Economic Studies, taught race relations and institutional racism at George Washington University, and was a consultant on race relations for the W.K. Kellogg Foundation. From 1981 to 1997, he represented the Governors of the 13 Appalachian states at the Appalachian Regional Commission. Before that, he held several policy-making positions in the administration of then-West Virginia Governor John D. Rockefeller IV. His memoir, *My Black Family, My White Privilege: A White Man's Journey Through the Nation's Racial Minefield*, was published in 2012. He graduated from Queens College in New York City, where he was a leader in the 1960s civil rights struggles.

## Notes

^*^

https://www.nationalcollaborative.org/our-programs/americans-support-racial-healing-less-divisive-political-environment-and-an-accurate-portrayal-of-racism-in-schools/

^†^

https://www.kff.org/racial-equity-and-health-policy/issue-brief/disparities-in-health-and-health-care-5-key-question-and-answers/

^‡^

https://www.citigroup.com/global/insights/citigps/closing-the-racial-inequality-gaps-20200922

^§^

https://supreme.justia.com/cases/federal/us/403/217/
